# Epidemiological pathology of Aβ deposition in the ageing brain in CFAS: addition of multiple Aβ-derived measures does not improve dementia assessment using logistic regression and machine learning approaches

**DOI:** 10.1186/s40478-019-0858-4

**Published:** 2019-12-05

**Authors:** S. B. Wharton, D. Wang, C. Parikh, F. E. Matthews, C. Brayne, P. G. Ince, on behalf of the Cognitive Function and Ageing Neuropathology Study Group

**Affiliations:** 1 Sheffield Institute for Translational Neuroscience, University of Sheffield, 385A Glossop Road, Sheffield, S10 2HQ UK; 2 Institute for Health and Society, University of Newcastle, Newcastle upon Tyne, UK; 3 Institute of Public Health, University of Cambridge, Cambridge, UK

## Abstract

Aβ-amyloid deposition is a key feature of Alzheimer’s disease, but Consortium to Establish a Registry for Alzheimer's Disease (CERAD) assessment, based on neuritic plaque density, shows a limited relationships to dementia. Thal phase is based on a neuroanatomical hierarchy of Aβ-deposition, and in combination with Braak neurofibrillary tangle staging also allows derivation of primary age-related tauopathy (PART). We sought to determine whether Thal Aβ phase predicts dementia better than CERAD in a population-representative cohort (*n* = 186) derived from the Cognitive Function and Ageing Study (CFAS). Cerebral amyloid angiopathy (CAA) was quantitied as the number of neuroanatomical areas involved and cases meeting criteria for PART were defined to determine if they are a distinct pathological group within the ageing population. Agreement with the Thal scheme was excellent. In univariate analysis Thal phase performed less well as a predictor of dementia than CERAD, Braak or CAA. Logistic regression, decision tree and linear discriminant analysis were performed for multivariable analysis, with similar results. Thal phase did not provide a better explanation of dementia than CERAD, and there was no additional benefit to including more than one assessment of Aβ in the model. Number of areas involved by CAA was highly correlated with assessment based on a severity score (*p* < 0.001). The presence of capillary involvement (CAA type I) was associated with higher Thal phase and Braak stage (*p* < 0.001). CAA was not associated with microinfarcts (*p* = 0.1). Cases satisfying pathological criteria for PART were present at a frequency of 10.2% but were not older and did not have a higher likelihood of dementia than a comparison group of individuals with similar Braak stage but with more Aβ. They also did not have higher hippocampal-tau stage, although PART was weakly associated with increased presence of thorn-shaped astrocytes (*p* = 0.048), suggesting common age-related mechanisms. Thal phase is highly applicable in a population-representative setting and allows definition of pathological subgroups, such as PART. Thal phase, plaque density, and extent and type of CAA measure different aspects of Aβ pathology, but addition of more than one Aβ measure does not improve dementia prediction, probably because these variables are highly correlated. Machine learning predictions reveal the importance of combining neuropathological measurements for the assessment of dementia.

## Introduction

Aβ amyloid deposition is widely accepted for decades as central to AD pathology, based on the amyloid cascade hypothesis as an upstream event [39]. However, population-based studies, including the United Kingdom-based Cognitive Function and Ageing Study (CFAS) have shown a limited relationship between histological Aβ plaque deposition and dementia status due to overlap in Aβ burden between demented and non-demented individuals, particularly in the oldest old [27, 33, 37].

Traditional assessment of cerebral Aβ load according to the Consortium to Establish a Registry for Alzheimer’s Disease (CERAD) standardised neuropathology protocol, proposed nearly 30 years ago, is based on plaque density, particularly neuritic plaques, in cortical areas [29]. Additional neuropathological measures have been proposed to assess Aβ. Thal phase provides a more finely graded assessment of Aβ-deposition, based on a proposed hierarchical progression of Aβ through neuroanatomical areas [42], analogous to the “Braak” method for assessment of neurofibrillary tangles (NFT) [7]. Thal phase, based on the detection of immunopositive amyloid in cortical and subcortical areas, distinguishes 5 phases based on progressive deposition of amyloid in neocortex (1), allocortex / limbic (2), diencephalon/basal ganglia (3), brainstem/midbrain (4), cerebellum (5). The assessment does not distinguish between compact/fibrillary and diffuse amyloid deposits. Thal phase, combined with CERAD neuritic plaque score and Braak NFT staging are incorporated into current National Institute on Aging recommendations for neuropathological assessment of dementia cases to derive the ABC score [31]. BrainNet Europe validated Thal phase and showed it can be applied consistently across European centres and assessors [1]. The Thal scheme also includes a qualitative assessment of the presence of cerebral amyloid angiopathy (CAA) in leptomeningeal and cortical vessels. CAA type 1 implies capillary amyloid is present, with or without staining in larger vessels. In CAA type 2 capillary amyloid is at most a minor component [42].

Integration of Thal Aβ and Braak NFT staging identified a putative sub-group of cases with mesial temporal tau pathology, up to Braak NFT stage IV, and little Aβ pathology, designated as “Primary age-related tauopathy (PART)”. Those cases having up to Braak stage IV but no Aβ pathology (Thal stage 0) are designated PART-definite and those or with mild Aβ pathology (Thal stages I-II) are designated PART-possible [10]. Whilst these distinctions can be pathologically defined and staged, debate remains as to whether PART is a distinct age-related entity or forms part of the Alzheimer pathology spectrum [11]. PART is associated with older age at death and lower cognitive scores [10], but its relationship to dementia and its natural history in unselected elderly populations is uncertain. Tau pathology may also occur in astrocytes in the ageing brain, particularly in mesial temporal structures [22, 38]. This includes various morphologies, including thorn-shaped astrocytes and granular or fuzzy astrocytes, and appears to be predominantly 4R tau. The assessment of this age-related tauopathy has recently been harmonized into the entity of aging-related tau astrogliopathy (ARTAG) [18, 19], though its relationship to other older age pathologies remains to be fully defined.

We aimed to investigate the variability of Aβ pathology in a population-representative neuropathology cohort and the relationship of Aβ-derived staging measures to dementia. We investigated the validity of Thal phase in a population-based cohort, hypothesising that inclusion of Thal Aβ phase would better predict dementia compared with CERAD score and improve pathological models of dementia. We explored quantitative assessment of CAA and determined whether cases meeting criteria for PART are a distinct pathological group within the ageing population.

We explored whether newer neuropathological measures improve the performance of Aβ measures for prediction of dementia. Specifically, we sought to: i. determine the validity of Thal phase in a population-representative cohort; ii. conduct statistical modelling and machine learning prediction to test whether inclusion of Thal Aβ phase offers better dementia prediction than CERAD score; iii. Provide quantitative assessment of CAA and determine its relationship to parenchymal Aβ and dementia; iv. identify cases meeting criteria for PART to determine whether they can be separated as a distinct pathological grouping within the ageing population and to determine the relationship to ARTAG.

## Methods

### Cohort

Tissue was used from the Newcastle and Cambridge centre sub-cohorts of CFAS (*n* = 186), as in our previous study [45]. Use of complete CFAS sub-cohorts maintains the population-representative nature of the study, without case pre-selection. Neuropathological lesions, including CERAD plaque score, Braak NFT, BrainNet Neuropil thread stage, ARTAG, vascular pathology and the presence of microinfarct stage were also previously assessed [16, 27, 33, 44, 45]. Hippocampal tau NFT stage, based on the method of Lace et al., was available on 94 cases from the Cambridge cohort [23]. Dementia status at death was established as present, absent, or uncertain, on the basis of AGECAT algorithm, death certification and Retrospective Informant Interview (RINI) [26, 32, 37, 44]. The study was undertaken with ethical approval from a UK Multicentre Research Ethics Committee (10/H0304/61).

### Neuropathological methods

Immunohistochemical detection of Aβ in formalin-fixed, paraffin-embedded sections (5 μm) used a standard avidin-biotin complex (ABC) method. Sections were deparaffinised, rehydrated to water and endogenous peroxidase activity quenched by placing the sections in 0.3% H_2_O_2_/methanol for 20 min at room temperature (RT). After antigen retrieval (0.01 M tri-sodium citrate pH 6.5, microwave 10 min) sections were subjected to formic acid pre-treatment for 60 min at RT. Following incubation with 1.5% normal serum for 30 min at RT, the sections were incubated with anti-Aβ (Clone 6F/3D; DakoCytomation, UK) at the optimal antibody dilution of 1:200 for 60 min at RT. To visualise antibody binding, the horse-radish peroxidase avidin biotin complex was used (Vectastain Elite kit, Vector Laboratories, UK) with 3,3′-diaminodenzidine (DAB) as the chromagen (Vector Laboratories, UK; brown) and lightly counterstained with Mayer’s haematoxylin. Negative controls, either omission of the primary antibody or isotype controls, were included in every run.

Assessment of Aβ phase was performed according to the Thal scheme, and BrainNet Europe approach [1, 42], based on the assessment of parenchymal deposits in: frontal, temporal, parietal and occipital cortex, temporal cortex adjacent to hippocampus (phase 1); hippocampus and cingulate gyrus (phase 2); striatum and basal forebrain (phase 3), midbrain central grey matter and substantia nigra (phase 4); cerebellum (phase 5). For many of the cases used in this study, basal forebrain was not well represented so that assessment of phase 3 was essentially dependent on striatal Aβ-deposition. Cytoplasmic Aβ was discounted for assessment of phase. Neurofibrillary tangles were assessed by Braak stage [6]. Plaques were assessed using the CERAD method [29], and for modelling, the maximum cortical neuritic plaque score was used.

We modified assessment of CAA to provide finer detail of the severity of CAA deposition. In addition to recording type I and II as in the scheme [42], we used two measures to further refine assessment of the burden of CAA: i. The number of anatomical areas involved from all the areas in the sampling set to obtain a measure of extent (number of areas out of 9 maximum); ii. The severity of leptomeningeal and parenchymal vascular amyloid in four neocortical areas according to the method of Love et al.: (score 1 - segmental involvement of vessels; score 2 - circumferential involvement; score 3 - widespread circumferential involvement, separately for parenchymal and leptomeningeal vessels [25]). Scores for leptomeningeal and parenchymal amyloid were summed in the four areas, giving a severity score out of maximum of 24.

Definite PART was defined as Aβ stage 0/Braak NFT stage I-IV and possible PART as Aβ stage 1–2/Braak NFT stage I-IV [10].

### Statistical analysis

Statistical analyses were performed using IBM SPSS v24 and RStudio v1.14. The Kolmogorov-Smirnov test was used to assess variables for a normal distribution. Differences in scores between 2 groups were assessed by Mann Whitney for unpaired data, and Wilcoxon signed ranks for related data. Correlation analysis used Spearman’s rho (ρ). Comparisons between multiple groups, with related data, were made using Friedman’s ANOVA for non-parametric data. Chi-square was used for categorical comparisons. Interactions between age, Thal and Braak stages were assessed using ANOVA.

For group comparisons, Braak NFT stages were divided into entorhinal (0-II), limbic (III-IV) and isocortical stages (V-VI). PART-definite (PART-d) cases were defined as those without amyloid and with Braak NFT stage I-IV. Part-possible (PART-p) cases were defined as Braak NFT stages I-IV and Thal phases 1–2 [10]. To compare PART with cases showing greater Aβ-pathology that may be more typical of AD-neuropathology progression, a PART-comparison group was defined with Braak NFT stages I-IV, but with Thal phases 3–5 (designated PART-c). See Fig. 5a for representation of these groups. For age groups, cases were divided into those individuals who were 79 yrs. or less at death (*n* = 39), a middle group of 80-89 yrs. (*n* = 83), and the oldest group, 90 yrs. and above (*n* = 63). Differences between age as a continuous variable and PART classes was assessed using the Wilcoxon test. All tests were two-tailed and significance was set at *p* < 0.05 except that, in cases where we compared all five neuropathological features, an alpha threshold for significance of 0.01 for significance after Bonferroni correction was used.

All cases were included for assessment of relationships between pathological variables, but cases where dementia was uncertain (i.e. could not be established from our algorithmic approach) were excluded from analyses in relation to dementia status.

### Predictive modelling using neuropathological features

In order to measure the ability of each classifier to generalise to some independent set, the out of bootstrap method was used. Samples were partitioned into a training set (75% of samples) and a validation set (25% of samples). The classifier was then trained on the training set, and performance evaluated on the validation set. This process was repeated by resampling the patients 1000 times with replacement to generate multiple test sets. The mean and standard deviation of the classification accuracy based on the predicted and observed clinical dementia diagnosis were computed for each sample. Data from CFAS was cleaned using pandas with certain factors, such as age groups being one hot encoded. Next we applied three different yet classical machine learning approaches for predicting dementia; logistic regression, decision tree and linear discriminant analysis (LDA) [12, 30, 46].

Logistic regression parameterises a sigmoid function to separate two classes of data. In this paper, the two classes were people with dementia and those without, with an odds ratio for a risk factor estimated. Logistic regression estimates the probability of having one of the two outcomes, is often used for this type of classification and it is relatively interpretable, not very computationally intense and very common. The logistic regression classifier was implemented using sklearn.linear_model package in the Python scikit-learn library.

Decision trees can be visualised as a flow chart or a tree structure where a node in a tree represents some feature and a branch is representative of a decision rule. The white box nature of decision trees makes them particularly well suited to applications where the understanding of the working of the classifier are essential. In addition to this, they are well suited to high dimensional data and, unlike the logistic regression model, are nonparametric. The decision tree class from the sklearn.tree package was used to implement the decision tree.

Linear discriminant analysis models the distribution of the risk factors separately in each dementia class, and then it uses Bayes theorem to estimate the probability of a linear combination of features from the risk factors being representative of a dementia class. One advantage of linear discriminant analysis over logistic regression is that it is more stable for smaller data sets and parameters estimates tend to be more stable for classes that are more distinctly separated. LDA has the assumption of normality in the independent variables. The Kolmogorov-Smirnov (KS) test was used to assess normality for these variables. The classifier was implemented using the LDA class from sklearn.discriminant_analysis.

## Results

### Characteristics of studied sample

The study cohort included 186 individuals from the Cambridge (*n* = 117) and Newcastle (*n* = 69) centres (age range 74 to 93 years at death). The demographics of the cohort are shown in Table 1. Dementia at death was present in 58% (107) of individuals and 38% (70) did not have dementia. Dementia status was uncertain for 9 individuals.

**Table 1:** Demographic and cognitive profile according to clinical dementia status

	No Dementia (*n* = 70)	Dementia (*n* = 107)	Unknown (*n* = 9)
Women^a^	37 (53%)	72 (67%)	4 (44%)
Age at death (years)^b^	84 (76–89)	89 (84–93)	84 (74–87)
Years since last cognitive assessment^b^	6 (3–10)	6 (3–10)	11 (5–14)
MMSE at last assessment ^b^	25 (21–24)	20 (17–24)	25 (24–25)

^a^ n (%)

^b^ median (inter-quartile range)

### Thal phase distribution across the cohort

**Fig. 1:**
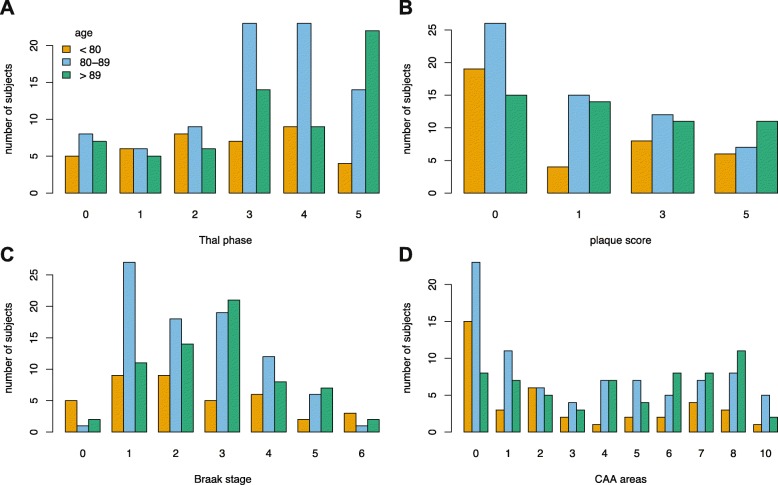
Histogram showing distribution of Aβ and tau pathology across the cohort subdivided into 3 age groups (*n* = 186), showing that older age groups show a shift to higher Thal phases; this is less apparent with other measures. **a** Thal phase, **b** CERAD neuritic plaque score, **c** Braak neurofibrillary tangle stage, **d** number of areas with CAA

The Newcastle and Cambridge subcohorts showed a similar distribution of Thal Aβ phases. Both had median scores of 3, with no significant difference in scores between centres (Mann Whitney *p* = 0.257). Agreement with the Thal scheme was excellent. Only 2 individuals deviated from the scheme; both had Thal phase 5 pathology in which staining in the stage 4 (midbrain) areas was not observed. More of the brains had pathology distributed towards the higher Thal phases, particularly for age groups 80-89 yrs. and 90 yrs. and above (Fig. 1a). CERAD plaque score, Braak NFT stage or CAA were not skewed towards higher scores at older ages (Fig. 1c-d).

### CAA

**Fig. 2:**
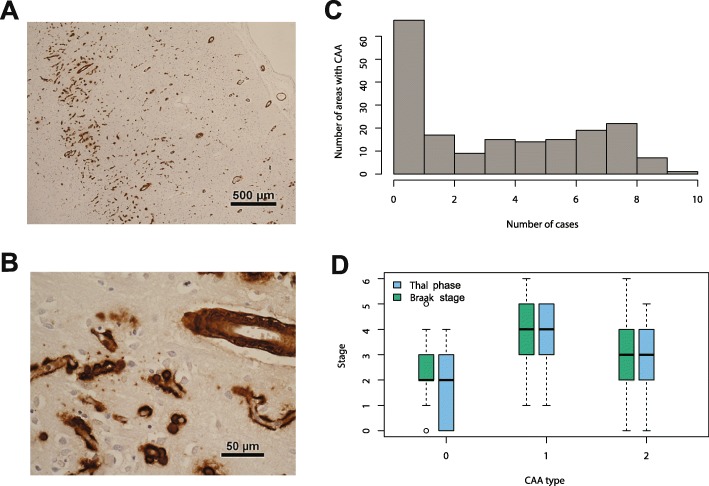
Cerebral amyloid angiopathy scores are positively skewed in the population, most individuals having a low score, and CAA type 1 is associated with high Braak stage and Thal phase. **a** and **b** Aβ deposition in occipital cortex showing deposition in leptomeningeal and parenchymal vessels, including capillaries (type I CAA). **c** Histogram showing distribution of CAA number of areas score. **d** Boxplots of Braak and Thal stages for cases with no CAA or with CAA types I and 2

CAA in any area was present in 75.3%. Of respondents with CAA (*n* = 140), 48.6% (68) were CAA type I (Fig. 2a,b), and 51.4% (72) were type II. CAA was scored as present or absent in the 10 assessed anatomical areas. The distribution of the number of areas involved by CAA was positively skewed with more individuals having a lower score and a score of 0 being the most prevalent (Fig. 2c). This was so for all age groups (Fig. 1d). CAA was most frequently detected in occipital cortex (66% of individuals in the cohort) compared to frontal cortex (55%), temporal cortex (53%) and parietal cortex (53%).

Semi-quantitative severity of CAA involvement, assessed in parenchyma and leptomeningeal vessels in four neocortical areas [25] was higher in the leptomeninges than in parenchyma (Wilcoxon signed ranks *p* < 0.001). There was strong correlation between the number of areas with CAA and severity of CAA as measured by the sum of the scores of vascular involvement in parenchymal and leptomeningeal vessels (Spearman’s *p* < 0.001, r_s_ = 0.94), indicating that either measure could be used as a global-brain semi-quantitative measure of CAA. Therefore, in further analyses we used only the number of areas with CAA.

The number of areas with CAA correlated with both Thal Aβ phase (Spearman’s *p* < 0.001, r_s_ = 0.64) and Braak NFT stage (*p* < 0.001, r_s_ = 0.52). Thal phase differed by CAA, absent, type I and type II CAA (Kruskal Wallis *p* < 0.001), as did Braak stage (*p* < 0.001). For both Thal and Braak, higher scores were associated with CAA type I (Fig. 2d).

We next determined whether CAA was associated with cortical microinfarcts. The number of areas with CAA did not correlate with the number of areas with cortical microinfarcts (Spearman’s *p* = 0.1, r_s_ = 0.12), with subcortical lacunes (*p* = 0.29, r_s_ = − 0.08) or the total number of areas with microinfarcts (*p* = 0.42, r_s_ = 0.06). There were no associations between CAA and microinfarcts in frontal (Chi Square χ^2^ = 0.94 1df, *p* = 0.33), temporal (χ^2^ = 0.34 1df, *p* = 0.56), parietal (χ^2^ = 0.19 1df, *p* = 0.67) or occipital (χ^2^ = 0.76 1df, *p* = 0.39) cortex.

### Relationships of Aβ deposition to dementia

**Fig. 3:**
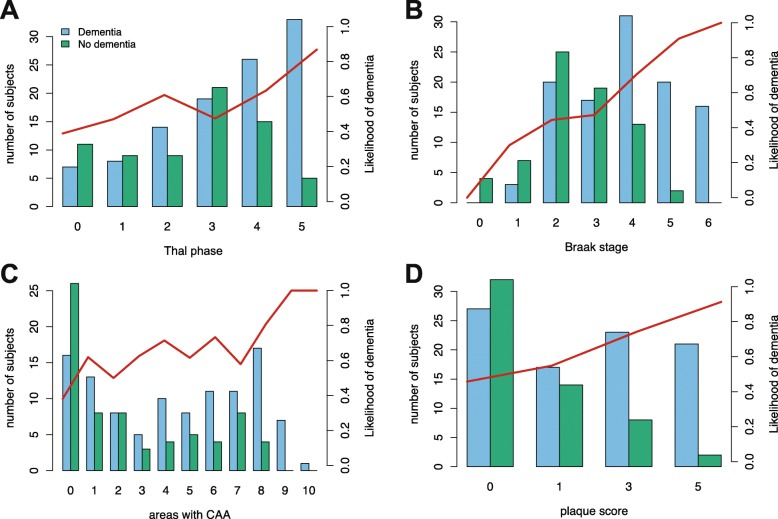
Number of individuals with and without dementia showing increasing proportions of individuals with increasing scores for the four pathological measures; **a** Thal phase, **b** Braak stage, **c** Number of areas with CAA, **d** neuritic plaque score. Red lines show likelihood of dementia

**Fig. 4:**
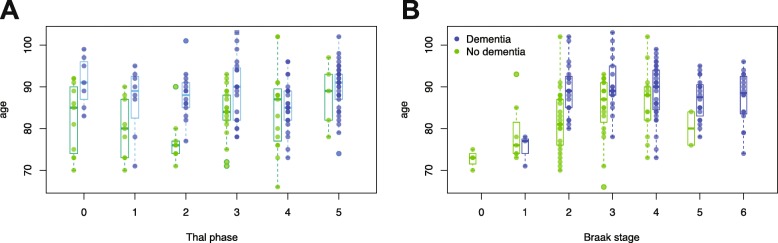
Age distribution for individuals with and without dementia for each neuropathological stage showing that individuals with dementia tend to older than those without for each Thal phase (**a**) and Braak stage (**b**)

We next investigated how Thal Aβ phase related to dementia. Thal phase measures neuroanatomical progression of Aβ rather than severity in only the neocortical areas and has a more granular scale. We hypothesised that Thal phase would better relate to dementia than CERAD plaque score, and provide additional information. The proportion of individuals with dementia increased with increasing Thal phase but this was also seen with plaque score and Braak NFT stage. The likelihood of dementia also increased in individuals with high CAA scores (Fig. 3). Importantly, cases of dementia were present in respondents with low burdens of Aβ, either by Thal or CERAD, and in those with low Braak NFT stage. The likelihood of dementia increased steadily with increasing Thal phase and Braak stage, except between stages 2 and 3. For both Thal and Braak, there were age-dependent relationships to dementia such that, for a given burden of pathology, individuals with dementia tended to be older those without (ANOVA *p* < 1.30e-06; Fig. 4), consistent with the role of age as a risk factor for dementia. However, there were low correlations between age and Thal phase and Braak stage for individuals with or without dementia (Spearman’s r_s_ < 0.37).

Accuracy of the prediction of dementia by these neuropathological measures was assessed in univariate analysis using logistic regression (Table 2). As univariate predictors of dementia, plaque score, measures of CAA and Braak stage all performed slightly better than Thal phase; however, prediction accuracy could be much better when using a combination of factors.

**Table 2:** Univariate classifier (logistic regression) of dementia status with different neuropathological features. The cross-validated mean accuracy and error was computed. Plaque score, measures of CAA and Braak stage all performed slightly better than Thal phase as dementia predictors

Features of univariate classifier	Mean accuracy (+/− error)
Thal Phase	0.5957 (+/− 0.0040)
Plaque score	0.6166 (+/− 0.0048)
CAA Type	0.6243 (+/− 0.0046)
CAA Areas	0.6222 (+/− 0.0047)
Braak Stage	0.6179 (+/− 0.0051)
Age (One hot encoded)	0.6540 (+/− 0.0038)
Brain weight	0.5721 (+/− 0.0032)

### Thal phase compared to plaque score in multivariable prediction models

We then assessed the predictive value of Thal phase for dementia in comparison with other measures when combined with other factors. Logistic regression provide similar results to two machine learning approaches, decision tree and LDA. The model was constructed using the following variables: Age and brain weight; Thal phase; Plaque score; Braak stage; CAA score. The model gave predictive accuracy for dementia of 0.6773 (CI 0.674–0.6806) using logistic regression, 0.701 (0.6967–0.7053) for decision tree and 0.6834 (0.6799–0.6869) for LDA. When each parameter was dropped singly from the model, age and brain weight had the largest effect. The neuropathological parameters each had similar effects (Table 3). The effects of Thal compared to other Aβ measures was assessed further by comparing Thal phase, CERAD score or CAA score when singly added to a model comprising age, brain weight and Braak score (Table 4). Adding any of these Aβ-related parameters gave a similar prediction performance compared to the best performing multivariable model (Accuracy = 0.7026, +/− 0.0048). This observation was consistent for the three types of predictive algorithms used.

**Table 3:** Classification of dementia status with different algorithms and different combinations of neuropathology features. Prediction accuracy and standard error are listed for each feature when it is omitted from the multivariable classifier. Age and brain weight had the largest effect when dropped from the model, whilst the neuropathological parameters each had similar effects

Classifier Type	Age & Brain weight	Thal Phase	Plaque score	Braak Stage	CAA	No features omitted
Logistic Regression	0.6327 (+/− 0.0035)	0.6774 (+/− 0.0033)	0.6773 (+/− 0.0033)	0.6776 (+/− 0.0033)	0.6785 (+/− 0.0034)	0.6773 (+/− 0.0033)
Decision Tree	0.6327 (+/− 0.0040	0.6997 (+/− 0.0045	0.6929 (+/− 0.0045	0.7026 (+/− 0.0048	0.7011 (+/− 0.0047	0.7010 (+/− 0.0043)
LDA	0.6344 (+/− 0.0032)	0.6763 (+/− 0.0035)	0.6709 (+/− 0.0034)	0.6738 (+/− 0.0034)	0.6760 (+/− 0.0.0036)	0.6834 (+/− 0.0035)

**Table 4:** Comparison of CAA, Thal phase and plaque score importance in classifiers that also use age, brain weight and Braak stage. Adding any one of the three Aβ-related parameters gave a similar prediction performance compared to the best performing multivariable model. Accuracy and confidence intervals are listed for dementia prediction when each neuropathology feature is omitted

Classifier (including age, brain weight and Braak score)	CAA	Thal Phase	Plaque score
Logistic regression	0.6756 (+/− 0.0032)	0.6885 (+/− 0.0036)	0.6741 (+/− 0.0030)
Decision Tree	0.6798 (+/− 0.0042)	0.6940 (+/− 0.0043)	0.6980 (+/− 0.0044)
LDA	0.6705 (+/− 0.0033)	0.6748 (+/− 0.0034)	0.6737 (+/− 0.0034)

### The relationship of Thal phase to Braak stage, and characteristics of PART in the cohort

**Fig. 5:**
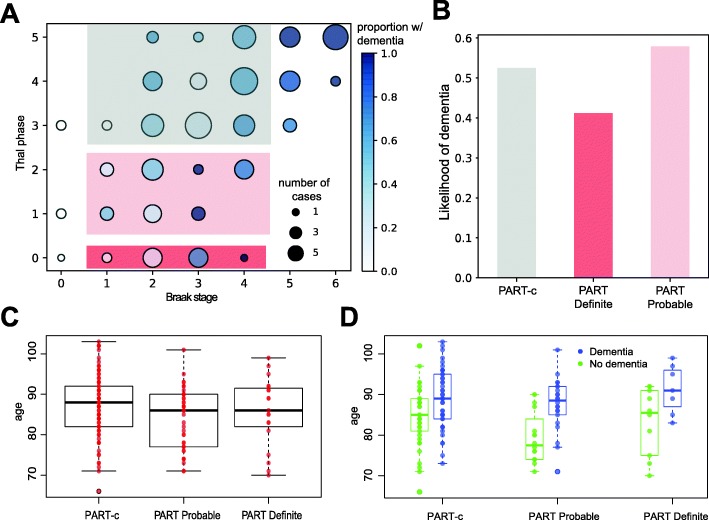
**a** Schematic showing NFT vs Aβ progression, with a main sequence of cases showing co-progression of the two AD neuropathologies. Cases can be defined showing NFT (Braak I-V) but no Aβ pathology corresponding to PART-d (red box), or with low levels of Aβ corresponding to PART-p (pink box). A comparison group, designated PART-c with similar Braak stage but more Aβ (Braak I-IV/Thal 3.-5) can be defined as those with more expected levels of Aβ for typical AD neuropathological progression (blue box). **b** likelihood of dementia for PART definite and probable cases and for the comparison PART-c group. **c** Overall age distribution of PART groups. **d** Age distribution according to dementia status for each of the PART groups

Thal Aβ phase correlated with Braak NFT stage (Spearman’s *p* < 0.001, r_s_ = 0.63). We identified 19 PART-definite cases (10.2%; Thal phase 0 / Braak NFT stage I-IV) and 39 PART-possible cases (21%; Thal phases 1–2 / Braak NFT stages I-IV), combined as 58 (31.2%) PART-all cases (Fig. 5).

To further define the nature of cases satisfying PART criteria in a population setting, we compared PART-definite (PART-d) and PART-all (PART-a) groups to those with the same Braak NFT stages (I-IV), but with the higher burdens of Aβ-deposition that might be more expected for typical AD neuropathological progression (PART-c) to determine whether PART cases show distinct features from cases with greater amyloid burden.

We hypothesised that the oldest individuals may develop mesial temporal tau formation with little amyloid. However, neither PART-d nor PART-a groups were older that the PART-c group (Wilcoxon test *p* > 0.67; Fig. 5c). PART-d showed a lower likelihood of dementia to the PART-c group, possibly due to the absence of associated Aβ, by definition, in PART-d. Both PART-d and PART-c group control groups showed that individuals with dementia tended to be older (Wilcoxon test *p* < 0.01; Fig. 5d).

We then investigated whether PART cases have more CAA compared to low Braak non-PART cases, because of greater clearance of Aβ into vessels. However, this was not the case; the number of anatomical areas with CAA was higher in the PART-c group than in either PART-d (Wilcoxon rank sum *p* < 0.001) or PART-a (*p* < 0.001).

We next investigated whether PART is associated with ARTAG, another age-related tauopathy characterised by various forms of glial tau, most commonly thorn-shaped astrocytes (TSA) [18]. If PART is an age-related tauopathy, a relationship to TSA might be expected. 63% of PART-d cases had TSA compared to 38% of PART-c (Chi Square χ^2^ = 3.98 1df *p* = 0.046). PART-a cases did not show greater TSA than the PART-c group (χ^2^ = 0.47 1df *p* = 0.49). The number of areas with TSA was greater in PART-d compared to the PART-c group (Mann Whitney *p* = 0.048), but there was no difference between the PART-a and PART-c groups (*p* = 0.43).

We hypothesised that, if PART represents a specific mesial temporal tauopathy PART cases would show more severe mesial temporal tau pathology than the PART-c group. We therefore determined whether PART cases had more hippocampal tau in a subcohort for which we had previously staged hippocampal tau (*n* = 93) [23]. PART-d (Mann Whitney *p* = 0.35) did not show a higher hippocampal tau load than the PART-c group. PART-a showed a significant difference in hippocampal tau stage to the PART-c (*p* = 0.014); however, contrary to our hypothesis the hippocampal stage was lower in the PART-a group so that PART is not associated with higher levels of hippocampal tau.

## Discussion

### Main study findings

Staging of Aβ based on neuroanatomical progression offers an alternative approach to semi-quantitative assessment of Aβ plaques, and comparison with neurofibrillary tangle staging allows further definition of the relative variation in Aβ and tau pathology between individuals. In this study, we show that Thal phase assessment is applicable to the continuum of amyloid burden found within a population-derived cohort. Logistic regression and machine learning approaches gave similar results for multivariable analysis to show that Thal phase does not provide a better explanation of dementia than the CERAD score. Assessment of CAA based on the number of anatomical areas involved, correlates with assessment of CAA based on severity, whilst the presence of capillary involvement (CAA type I) is associated with higher burdens of parenchymal Aβ and tau pathology. Thal phase allows ascertainment of PART cases within the cohort with a frequency of about 10% (for PART-d). Individuals with PART reflect population variation in pathology, rather than a discrete group within the population. PART individuals were not older than the comparison group of individuals with higher Aβ for the same Braak stages (PART-c; Braak I-IV/Thal 3–5), although PART was associated with ARTAG, suggesting a relationship with age-related mechanisms. PART cases also did not show a higher likelihood of dementia at death, although both PART and the PART-c with dementia at death were older than individuals without dementia.

### Study limitations

There are a number of limitations to this study. The study is focused on the variation and comparison of different measures of Aβ-deposition in a population-derived sampled. This study does not include a comprehensive analysis of the various pathologies, such as α-synuclein, TDP-43 and vascular pathology, that may coexist in the ageing brain, which may affect the behaviour of Aβ-derived measures in modelling. Although based on a well-characterised population-representative cohort that was not pre-selected for clinical diagnosis [8, 44], the sample used for this study was limited to 186 participants. Using the Cambridge and Newcastle centre sub-cohorts maintained the population-representative basis of the study. The assessment of cognitive status was based on the presence or absence of dementia at death [8, 37, 44]. The approach taken in CFAS is reliable for dementia assessment in the population context, but the study did not assess the relationship to mild cognitive impairment or to atypical dementia syndromes. The study was focused on the Aβ-deposition detectable by conventional immunohistochemistry in formalin-fixed, paraffin embedded tissue. It did not assess lower molecular weight soluble aggregates, which may also relate to dementia [17, 28, 39], so that it gives an incomplete view of the role of Aβ in dementia. However, the approach used is appropriate for the study aims of assessing morphology-based staging of Aβ, which are currently the basis for complete clinicopathological diagnosis. Incorporating measures of additional Aβ species would be a valuable further step. This study did not assess *APOE* genotype or tau haplotype, which may vary with AD and tangle-predominant dementia [36]. The study, based on autopsy samples is cross-sectional and assessment of dementia necessarily retrospective. Longitudinal imaging studies of cerebral Aβ suggested that its accumulation is early in relation to cognitive decline, and may predict progression, whereas further cognitive decline is less related to Aβ burden [43]. Our study could not detect such interactions.

### Thal phase and dementia

Thal phase [42], like Braak stage for NFT [7], is based on the concept of progressive, hierarchical recruitment of brain areas. The Thal scheme showed excellent validation of this approach at the population level in this cohort. The scheme is easy to apply and offers a more finely granular Aβ assessment than the CERAD score [29].

In univariate analysis, however, Thal phase was less predictive for dementia than CERAD and multivariable analysis showed no advantage for Thal phase compared to CERAD score, and its inclusion did not improve the model when CERAD score was already included. Both the logistic regression and machine learning approaches gave similar results. This supports findings in other studies. Serrano-Pozo et al., in 192 subjects in the longitudinal cohort from the Alzheimer’s Disease Centers, found that Thal phase did not significantly associate with Clinical Dementia Rating Sum of Boxes (CDR-SOB) or Mini-Mental State Examination scores and did not improve prediction of dementia, whereas CERAD did associate with CDR-SOB [40]. Boluda et al. found a lack of correlation between CERAD and Thal phase, except at higher scores, but found that Thal phase was more predictive of dementia than CERAD, although this study used AD cases and controls and so may not have reflected the continuum of pathology seen in a population [4]. Braak NFT stage correlates with measures of clinical course in AD, including final MMSE score, but Thal phase does not after accounting for Braak [34]. The CERAD method, whilst confined to the neocortex, assesses neuritic plaques so that it may be detecting more functionally significant Aβ-deposition compared to inclusion of diffuse deposits using Thal phase. Thal phase and CERAD score reflect different aspects of Aβ as supported by the relationship of age with higher Thal phases but not CERAD score, so that the increasing neuroanatomical spread of diffuse Aβ is more related to ageing. While Thal phase is related to the likelihood of dementia, it does not improve prediction of dementia compared to CERAD score in the diagnostic assessment of cases. Including Thal phase, however, allows assessment of variation in relative abundance of Aβ to tau and the definition of subgroups such as PART cases.

Thal phase is highly correlated with Braak NFT stage, although the anatomical hierarchies differ. For example, Thal phase 1 reflects neocortical involvement by Aβ as the earliest stage, whereas NFT formation shows earliest involvement of entorhinal and hippocampal structures, and the neocortex is involved at later stages [6, 7]. Some authors suggest that tau pathology begins in brainstem nuclei, although not all studies agree [3, 41]. Both the Thal and Braak schemes are operationalised in the context of a continuum of the respective pathologies. However whilst Aβ and NFT tend to progress together, the differing neuroanatomical hierarchies remain unexplained by current theories of the amyloid cascade hypothesis.

Age and brain weight had a larger effect in the multivariable model than any of the individual neuropathological parameters as previously reported by CFAS [27] and, for most of the individual Thal phases and Braak stages, individuals with dementia were older than those without. This was also true for each of the PART (−d and –p) groups and the PART-c, comparison, group. Age is the largest risk factor for dementia and ageing itself may impair cells through multiple mechanisms [24]. This supports the concept of aging mechanisms as additional contributors to dementia and the importance of age as a parameter in models of late-life dementia. .

### CAA

CAA was present in around 75% of cases, and of those with CAA approximately half were type I CAA, in which there is also capillary involvement. This prevalence is higher than in some studies. CAA prevalence has been reported at 53.8% in an unselected autopsy series [21] and 44.1% in the Honolulu-Asia Aging Study (HAAS), a population representative study of male Japanese migrants to Hawaii [35]. The prevalence estimate reported here is more similar to a combined community-based sample of 1113 individuals in the Rush Memory and Aging Study and the Religious Orders Study (78.9%) [5]. As in previous studies [21, 35], the highest regional involvement by CAA is in the occipital cortex.

We found that quantifying the number of areas with CAA correlated well with assessments of CAA severity based on extent of vascular involvement in neocortical areas [25], and so used this in our analyses. CAA correlated with Thal phase. As in previous studies, the presence of CAA was associated with greater burdens of amyloid and tau with a stronger association with Aβ [2, 5, 13, 21, 35], and capillary involvement (CAA type 1) was associated with higher levels of AD pathologies.

Previously published studies conflict on the relationship between CAA and cortical microinfarcts. CAA was found to be associated with microinfarcts in the allocortical region in an unselected, but not population-representative, autopsy series [15] and CAA correlated with microinfarcts in a small series of vascular dementia cases [14]. In HAAS, CAA was not associated with ischaemic or haemorrhagic lesions [35]. Previous work in CFAS did not find a relationship between CAA and microinfarcts [16], but CAA assessment in that study was based on a version of CERAD assessment of the case prior to systematic adoption of CAA into the CERAD protocol. Using a more rigorous assessment, we confirm that microinfarcts do not show a statistical relationship to CAA in this cohort. We did not confirm the specific association found in occipital cortex [20], although this could be an effect of small numbers with microinfarcts in each cortical area. Further data on the relationship of CAA to specific forms of vascular brain pathology are required to resolve these inconsistencies.

CAA is associated with dementia, mild cognitive impairment and more focal measures of impaired cognition such as perceptual speed and episodic memory [2, 5, 9, 15]. These effects may be separate from the effects of AD neuropathological change. In HAAS, CAA did not alter the risk for dementia, but there was a significant interaction between CAA and AD neuropathological change so that cognition in men with both was worse than in those with either alone [35]. Univariate analysis in the present study showed that the number of areas involved by CAA was a predictor of dementia of similar magnitude to parenchymal Aβ scores, but in multivariable modelling it contributed little additional predictive information. Binary cognitive stratification in our study was limited to dementia status and we cannot exclude a contribution of CAA to mild cognitive impairment or more specific cognitive domains.

### PART

Within the continuum of Aβ and tau pathology we defined cases with neurofibrillary tangles (up to Braak stage IV) but with absent or low level Aβ deposition that correspond to the definition of PART [10]. We also defined a comparison group, PART-c, with a higher burden of Aβ-pathology (as may be expected from cases more typically on an AD pathological trajectory). PART-d cases (those with Thal phase 0) were present at a frequency of about 10% in our population sample. Whether PART falls within the AD spectrum or is a distinct age-related tauopathy is unresolved [11]. Because Aβ and NFT burden across the population lie on continuums, PART may not be patho-aetiologically distinct but merely represent cases lying off the main sequence of AD neuropathology progression (Fig. 5). In this study there is no evidence to define this pathological constellation as a distinct disease group. It is also possible to select a group with Aβ pathology but with no or minimal tangle formation who also demonstrate a spectrum of clinical outcomes.

Defining entities like PART, with varied relative burdens of the different AD neuropathological lesions is important to appreciate the heterogeneity, and possible implications, of late-life AD neuropathologic change. Low parenchymal Aβ in PART is not due to preferential distribution into vessels since CAA was higher in the PART-c group. Rather, the higher CAA in low-Braak stage cases with parenchymal amyloid reflects a correlation between parenchymal Aβ and CAA. It has been suggested that, as an age-related tauopathy, PART might be associated with more severe mesial temporal tau [11]. The present data do not demonstrate a higher hippocampal tau stage [23] in PART, and PART cases were not older than PART controls. We did find an association between PART and thorn-shaped astrocytes, which are a feature of age-related tau astrogliopathy (ARTAG) [18, 45], so that ageing mechanisms likely influence PART pathogenesis.

### Conclusions

More detailed approaches to staging are helpful to appreciate and stratify heterogeneity within the spectrum of late-life AD neuropathological change. Ultimately such stratification is only useful if it can contribute to enhanced understanding of dementia risk and pathogenetic cascades. More granular characterisation is potentially important to assess the impact of brain ageing and genetic factors on pathological heterogeneity and cognitive outcomes. The incorporation of advanced bioinformatics approaches in this study shows that combining Thal phase, CERAD score and improved semi-quantitative assessment of CAA, assessing different aspects of Aβ pathology, does not improve dementia prediction, possibly because such variables are correlated. Selection of specific amyloid assessment protocols for diagnostic and research purposes clearly requires careful consideration but is not likely to generate significantly conflicting estimates of diagnostic categories between studies.

## References

[1] Alafuzoff I, Thal D, Arzberger T, Bogdanovic N, Al-Sarraj S, Bodi I, Boluda S, Bugiani O, Duyckaerts C, Gelpi E (2009). Assessment of β-amyloid deposits in human brain: a study of the BrainNet Europe Consortium. Acta Neuropathol.

[2] Arvanitakis Z, Leurgans S, Wang Z, Wilson R, Bennett D, Schneider J (2011). Cerebral amyloid angiopathy pathology and cognitive domains in older persons. Ann Neurol.

[3] Attems J, Thal D, Jellinger K (2012). The relationship between subcortical tau pathology and Alzheimer's disease. Biochem Soc Trans.

[4] Boluda S, Toledo J, Irwin D, Raible K, Byrne M, Lee E, Lee V, Trojanowski J (2014). A comparison of Aβ amyloid pathology staging systems and correlation with clinical diagnosis. Acta Neuropathol.

[5] Boyle P, Yu L, Nag S, Leurgans S, Wilson R, Bennett D, Schneider J (2015). Cerebral amyloid angiopathy and cognitive outcomes in community-based older persons. Neurology.

[6] Braak H, Alafuzoff I, Arzberger T, Kretaschmar H, del Tredici K (2006). Staging of Alzheimer disease-associated neurofibrillary pathology using paraffin sections and immunocytochemistry. Acta Neuropathol.

[7] Braak H, Braak E (1991). Neuropathological stageing of Alzheimer-related changes. Acta Neuropathol.

[8] Brayne C, McCracken C, Matthews F (2006). Cohort profile: the Medical Research Council cognitive function and ageing study (CFAS). Int J Epidemiol.

[9] Case N, Charlton A, Zwiers A, Batool S, McCreary C, Hogan D, Ismail Z, Zerna C, Coutts S, Frayne R (2016). Cerebral amyloid angiopathy is associated with executive dysfunction and mild cognitive impairment. Stroke.

[10] Crary J, Trojanowski J, Schneider J, Abisambra J, Abner E, Alafuzoff I, Arnold S, Attems J, Beach T, Bigio E (2014). Primary age-related tauopathy (PART): a common pathology associated with human aging. Acta Neuropathol.

[11] Duyckaerts C, Braak H, Brion J-P, Buee L, del Tredici K, Goedert M, Halliday G, Neumann M, Spillantini M, Tolnay M (2015). PART is part of Alzheimer’s disease. Acta Neuropathol.

[12] French B, Dawson M, Dobbs A (1997). Classification and staging of dementia of the Alzheimer type. A comparison between neural networks and linear discriminant analysis. Arch Neurol.

[13] Ganz A, Beker N, Hulsman M, Sikkes S, Scheltens P, Smit A, Rozemuller A, Hoozemans J, Holstege H (2018). Neuropathology and cognitive performance in self-reported cognitively healthy centenarians. Acta Neuropathol Comm.

[14] Haglund M, Passant U, Sjobeck M, Ghebremedhin E, Englund E (2006). Cerebral amyloid angiopathy and cortical microinfarcts as putative substrates of vascular dementia. Int J Geriatr Psychiatry.

[15] Hecht M, Kramer L, von Arnim C, Otto M, Thal D (2018). Capillary cerebral amyloid angiopathy in Alzheimer’s disease: association with allocortical/hippocampal microinfarcts and cognitive decline. Acta Neuropathol.

[16] Ince P, Minett T, Forster G, Brayne C, Wharton S (2017). Microinfarcts in an older population-representative brain donor cohort (MRC-CFAS): prevalence, relation to dementia and mobility, and implications for the evaluation of cerebral small vessel disease. Neuropathol Appl Neurobiol.

[17] Koss D, Jones G, Cranston A, Gardner H, Kanaan N, Platt B (2016). Soluble pre-fibrillar tau and β-amyloid species emerge in early human Alzheimer’s disease and track disease progression and cognitive decline. Acta Neuropathol.

[18] Kovacs G, Ferrer I, Grinberg L, Alafuzoff I, Attems J, Budka H (2016). Aging-related tau astrogliopathy (ARTAG): harmonized evaluation strategy. Acta Neuropathol.

[19] Kovacs G, Xie S, Lee E, Robinson J, Caswell C, Irwin D, Toledo J, Johnson V, Smith D, Alafuzoff I (2017). Multisite assessment of aging-related tau astrogliopathy (ARTAG). J Neuropathol Exp Neurol.

[20] Kovari E, Herrmann F, Gold G, Hof P, Charidimou A (2017). Association of cortical microinfarcts and cerebral small vessel pathology in the ageing brain. Neuropathol Appl Neurobiol.

[21] Kovari E, Herrmann F, Hof P, Bouras C (2013). The relationship between cerebral amyloid angiopathy and cortical microinfarcts in brain ageing and Alzheimer’s disease. Neuropathol Appl Neurobiol.

[22] Lace G, Ince P, Brayne C, Savva G, Matthews F, de Silva R, Simpson J, Wharton S (2012). Mesial temporal astrocyte tau pathology in the MRC-CFAS ageing brain cohort. Dement Geriatr Cogn Disord.

[23] Lace G, Savva G, Forster G, de Silva R, Brayne C, Matthews F, Barclay J, Dakin L, Ince P, Wharton S, On behalf of MRC-CFAS (2009). Hippocampal tau pathology is related to neuroanatomical connections: an ageing population-based study. Brain.

[24] Lopez-Otin C, Blasco M, Partridge L, Serrano M, Kroemer G (2013). The hallmarks of ageing. Cell.

[25] Love S, Chalmers K, Ince P, Esiri M, Attems J, Jellinger K, Yamada M, McCarron M, Minett T, Matthews F (2014). Development, appraisal, validation and implementation of a consensus protocol for the assessment of cerebral amyloid angiopathy in post-mortem brain tissue. Am J Neurodegen Dis.

[26] Marioni R, Matthews F, Brayne C (2011). The association between late-life cognitive test scores and retrospective informant interview data. Int Psychogeriatr.

[27] Matthews F, Brayne C, Lowe J, McKeith I, Wharton S, Ince P (2009). Epidemiological pathology of dementia: attributable-risks at death in the MRC cognitive function and ageing study. PLoS Med.

[28] McDonald J, Savva G, Brayne C, Welzel A, Forster G, Shankar G, Selkoe D, Ince P (2010). The presence of sodium dodecyl sulphate-stable Aβ dimers is strongly associated with Alzheimer-type dementia. Brain.

[29] Mirra S, Heyman A, McKeel D, Sumi S, Crain B, Brownlee L, Vogel F, Hughes J, Van Belle G, Berg L (1991). The Consortium to Establish a Registry for Alzheimer’s Disease (CERAD). Part II Standardisation of the neuropathologic assessment of Alzheimer’s disease. Neurology.

[30] Mofrad R, Schoonenboom N, Tijms B, Scheltens P, Visser P, Van der Flier W, Teunissen C (2019). Decision tree supports the interpretation of CSF biomarkers in Alzheimer’s disease. Alzheimers Dement.

[31] Montine T, Phelps C, Beach T, Bigio E, Cairns N, Dickson D, Duyckaerts C, Frosch M, Masliah E, Mirra S (2012). National Institute on Aging-Alzheimer’s Association guidelines for the neuropathologic assessment of Alzheimer’s disease: a practical approach. Acta Neuropathol.

[32] MRC-CFAS (1998). Cognitive function and dementia in six areas of England and Wales: the distribution of MMSE and prevalence of GMS orgnanicity level in the MRC CFA study. Psychol Med.

[33] MRC-CFAS (2001). Pathological correlates of late-onset dementia in a multicentre, community-based population in England and Wales. Lancet.

[34] Murray M, Lowe V, Graff-Radford N, Liesinger A, Cannon A, Przybelski S, Rawal B, Parisi J, Petersen R, Kantarci K (2015). Clinicopathologic and ^11^C-Pittsburgh compound B implications of Thal amyloid phase across the Alzheimer’s disease spectrum. Brain.

[35] Pfeifer L, White L, Ross G, Petrovitch H, Launer L (2002). Cerebral amyloid angiopathy and cognitive function. The HAAS autopsy study. Neurology.

[36] Santa-Maria I, Haggiagi A, Liu X, Wasserscheid J, Nelson P, Dewar K, Clark L, Crary J (2012). The *MAPT* H1 haplotype is associated with tangle-predominant dementia. Acta Neuropathol.

[37] Savva G, Wharton S, Ince P, Forster G, Matthews F, Brayne C (2009). For the medical research council cognitive function and ageing study. Age, neuropathology, and dementia. N Engl J Med.

[38] Schultz C, Ghebremedhin E, del Tredici K, Rub U, Braak H (2004). High prevalence of thorn-shaped astrocytes in the aged human medial temporal lobe. Neurobiol Aging.

[39] Selkoe D, Hardy J (2016). The amyloid hypothesis of Alzheimer’s disease at 25 years. EMBO Mol Med.

[40] Serrano-Pozo A, Qian J, Muzikansky A, Monsell S, Montine T, Frosch M, Betensky R, Hyman B (2016). Thal amyloid stages do not significantly impact the correlation between neuropathological change and cognition in the Alzheimer disease continuum. J Neuropathol Exp Neurol.

[41] Stratmann Katharina, Heinsen Helmut, Korf Horst-Werner, Del Turco Domenico, Ghebremedhin Estifanos, Seidel Kay, Bouzrou Mohamed, Grinberg Lea T., Bohl Jürgen, Wharton Stephen B., den Dunnen Wilfred, Rüb Udo (2015). Precortical Phase of Alzheimer's Disease (AD)-Related Tau Cytoskeletal Pathology. Brain Pathology.

[42] Thal D, Rub U, Orantes M, Braak H (2002). Phases of Aβ-deposition in the human brain and its relevance for the development of AD. Neurology.

[43] Villemange V, Pike K, Chetelat G, Ellis K, Mulligan R, Bourgeat P, Ackermann U, Jones G, Szoeke C, Salvado O (2011). Longitudinal assessment of Aβ and cognition in aging and Alzheimer disease. Ann Neurol.

[44] Wharton S, Brayne C, Savva G, Matthews F, Forster G, Simpson J, Lace G, Ince P (2011). Epidemiological neuropathology: the MRC cognitive function and ageing study experience. J Alzheimer Dis.

[45] Wharton S, Minett T, Drew D, Forster G, Matthews F, Brayne C, Ince P (2016). Epidemiological pathology of tau in the ageing brain: application of staging for neuropil threads (BrainNet Europe protocol) to the MRC cognitive function and ageing brain study. Acta Neuropathol Commun.

[46] Zheng C, Xia Y, Pan Y, Chen J (2016). Automated identification of dementia using medical imaging: a survey from a pattern classification perspective. Brain Inform.

